# Driver behaviour data linked with vehicle, weather, road surface, and daylight data

**DOI:** 10.1016/j.dib.2016.12.036

**Published:** 2016-12-21

**Authors:** Odd André Hjelkrem, Eirin Olaussen Ryeng

**Affiliations:** Norwegian University of Science and Technology, Norway

## Abstract

In this data set, vehicle observations have been linked to data containing weather and road surface conditions. A total of 311 908 observations are collected and classified in categories of precipitation type, road status information, and daylight condition. The data is collected for a long period of time, so that several different weather situations are present, ranging from dry summer to adverse winter weather conditions.

**Specifications Table**

**Table:** 

Subject area	*Transport Engineering.*
More specific subject area	*Traffic flow, traffic safety*
Type of data	*Table,.csv file.*
How data was acquired	*Detection equipment, ephemeris, visual investigation.*
Data format	*Raw, analysed*
Experimental factors	*Raw data obtained from WIM-detectors, a weather station and a roadside camera.*
Experimental features	*Image classification performed after data collection.*
Data source location	*Latitude: 62.48780N, Longitude: 7.76104E.*
Data accessibility	*Data is included in this article.*

**Value of the data**

• *A unique collection of linked data sources offers a large number of factors available for studying vehicle behaviour.*
• *The data set include vehicles in both free flow and car-following state, so that the impact of both traffic and surroundings can be analysed.*
• *The vehicles observed are diverse in physical characteristics and driver behavior, allowing for studies of either specific vehicle types or the entire traffic flow.*
• *The weather situations observed range from normal conditions to adverse conditions, such as snow during night time on snow covered roads, facilitating studies of weather related driver behaviour.*

## 1 Data

This article describes a data set related to the article "Chosen risk level during car-following in adverse weather conditions" (O.A. Hjelkrem, E.O. Ryeng, 2016) [1]. A presentation of the available attributes in the data set (see Supplementary material.csv file) is shown in Table 1.

## 2 Experimental design, materials and methods

The vehicle and weather detection equipment are placed close to each other at a rural two-lane road in Norway. The speed limit at the site is 80 kph. The traffic level at the site is quite low, with an AADT of about 2000. Located about 15 km from the city of Åndalsnes, the road is an important route for road transport between Oslo and the west coast. About 10 km further east, there is a climb of about 500 m for 25 km, which is quite steep. Still, there are no viable alternatives for heavy vehicles, so the heavy vehicle percentage is about 10%. The data was recorded between March 21th 2012 and April 30th 2014, although not continuously.

### 2.1 Observations

The vehicle detectors measured speed, time, lane number, vehicle length, vehicle weight, and number of axles for each passing vehicle. From this data, it was possible to derive time gap and information about the lead and following vehicle.

The roadside weather station measured meteorological data every 10 min, including precipitation intensity, precipitation type, air temperature, relative humidity, wind speed and wind direction.

The weather station was also equipped with a camera which stored a picture of the road surface approximately every 10 min. These pictures were used to identify the road surface conditions. All pictures in the detection period were manually investigated to classify the road surface according to the categories ׳dry׳, ׳wet׳, ׳snow covered׳ and ׳visible tracks’ in snow, with the latter category meaning longitudinal bare strips on a surface otherwise covered by snow. The definition of snow cover is when the complete road surface is covered with snow.

Using the spatial position of the detector site and the point in time for each vehicle detection event, the lighting condition was found by looking up in an ephemeris, which is an almanac for the movements of the celestial bodies. The specific ephemeris used was the PyEphem module available for Python [2]. It can be used to determine the sunrise and sunset at a specific place and time on the planet. The lighting conditions were categorised into daylight, twilight and night. Twilight was defined as the time period one hour before and one hour after both sunset and sunrise.

### 2.2 Data join

The following routine was used for creating the data set:

1. Import vehicle observations into a PGSQL-database.
2. Assign time of day to each observation.
3. Import weather data to another PGSQL-database.
4. Join the two databases using the time of each vehicle event and each 10-min interval of weather data.
5. Add information about the preceding vehicle (ID, speed, weight and length).
6. Add information about road status information based on the manual classification of images.

## Figures and Tables

**Table 1:** Description and range of attributes in the data set, *N*=311 908.

Attribute	Data type	Range
*ID*	*Integer*	*19 to 794 438*
*Timestamp*	*Text*	*21.03.2012 to 30.04.2014*
*Vehicle length*	*Floating point*	*102 to 2981 *cm
*Lane*	*Integer*	*1 or 2*
*Vehicle speed*	*Floating point*	*0 to 169 *kph
*Vehicle weight*	*Floating point*	*0 to 69 548 *kg
*Number of axles*	*Integer*	*2 to 11*
*Validity code*	*Text*	
*Lead vehicle ID*	*Integer*	*19 to 794 436*
*Lead vehicle speed*	*Floating point*	*0 to 169 *kph
*Lead vehicle weight*	*Floating point*	*0 to 69 548 *kg
*Lead vehicle length*	*Floating point*	*102 to 2981 *cm
*Time gap*	*Floating point*	*0 to 530 331 *s
*Air temperature*	*Floating point*	*−13.6 to 24.8*
*Precipitation type*	*Text*	*Clear, Rain, Snow*
*Precipitation intensity*	*Text*	*None, Low 0–1 (*mm/10 min*), Moderate 1–5 (*mm/10 min*), High above 5 (*mm/10 min*)*
*Relative humidity*	*Floating point*	*16 to 97*%
*Wind direction*	*Integer*	*0 to 360* degrees
*Wind speed*	*Floating point*	*0 to 23.1 *m/s
*Road surface status*	*Text*	*Dry, Wet, Visible tracks, Snow covered*
*Time of day*	*Text*	*Daylight, Night, Twilight*
